# Arntl-induced upregulation of DUSP1 inhibits tumor progression in esophageal squamous cell carcinoma by inactivating ERK signaling

**DOI:** 10.1080/15384047.2024.2408042

**Published:** 2024-09-28

**Authors:** Jianjun Wang, Qifan Jia, Jingyao Sun, Sen Wu, Li Wei, Wenjian Yao

**Affiliations:** a Department of Thoracic Surgery, Henan Provincial People’s Hospital, People’s Hospital of Zhengzhou University, School of Clinical Medicine, Henan University, Zhengzhou, Henan, China; b Department of Thoracic Surgery, Zhengzhou University People’s Hospital, Henan Provincial People’s Hospital, Zhengzhou, Henan, China

**Keywords:** Esophageal squamous cell carcinoma, apoptosis, ARNTL, DUSP1, ERK

## Abstract

**Background:**

Esophageal squamous cell carcinoma (ESCC) is a primary histological type of esophageal carcinoma with high morbidity. Aryl hydrocarbon receptor nuclear translocator-like (ARNTL) is a circadian clock gene associated with the progression of multiple tumors. However, its roles and mechanisms in ESCC remain unknown.

**Methods:**

ARNTL expression was analyzed using TCGA database and detected using qRT-PCR, and ARNTL-related pathways were analyzed through GSEA. Cell functional behaviors were assessed in vitro by measuring cell viability, proliferation, and apoptosis. Cell growth in the murine model was investigated through xenograft model and immunofluorescence assays of PCNA and Ki67. The downstream targets of ARNTL were analyzed through sequencing and identified via luciferase report, ChIP, and RNA pull-down analyses. Dual-specificity protein phosphatase-1 (DUSP1) expression was analyzed using GEO datasets and measured using qRT-PCR and western blotting. Protein expression was examined via western blotting.

**Results:**

ARNTL expression was decreased in esophageal carcinoma and associated with histological types, and elevated expression of ARNTL repressed ESCC cell viability and proliferation and facilitated cell apoptosis. ARNTL upregulation reduced tumor cell growth in murine models and decreased PCNA and Ki67 levels. Furthermore, DUSP1 was downregulated upon ARNTL silencing in ESCC. ARNTL could bind and positively regulate DUSP1 transcription. Additionally, DUSP1 silencing reversed the influences of ARNTL upregulation on cell viability, proliferation, and apoptosis in ESCC cells. ARNTL attenuated the activation of the ERK signaling by decreasing ERK phosphorylation through upregulation of DUSP1.

**Conclusion:**

ARNTL hinders cell growth and contributes to cell apoptosis by inactivating ERK signaling through transcriptional upregulation of DUSP1 in ESCC.

## HIGHLIGHTS

• ARNTL is differentially expressed in ESCC and associated with cell apoptosis.

• ARNTL augments cell apoptosis.

• ARNTL increases DUSP1 transcription.

• ARNTL inhibits activation of the ERK signaling by upregulating DUSP1.

• DUSP1 silencing reverses the effects of ARNTL in esophageal squamous cell carcinoma.

## Introduction

Esophageal carcinoma ranks eleventh in incidence, with 510,716 new cases, and seventh in mortality, with 445,129 deaths worldwide.^1^ Esophageal squamous cell carcinoma (ESCC) is the most common histological subtype.^2^ Due to the lack of specific early diagnostic and prognostic biomarkers, the prognosis of ESCC remains poor.^3^ Its five-year survival rate is approximately 15–20%.^4^ Thus, exploring effective therapeutic targets for the diagnosis and treatment of ESCC is urgently required.

Several differentially expressed genes are documented in ESCC and play key roles in cancer development.^5^ Aryl hydrocarbon receptor nuclear translocator-like (*ARNTL*, also known as *BMAL1*) is a circadian clock gene that acts as a transcription factor regulating RNA transcriptional activation.^6,7^ ARNTL is reportedly involved in various disorders, including tumors, COVID-19, periodontitis, and skeleton disorders.^8–10^ Multiple reports have demonstrated the anti-tumor roles of ARNTL in human tumors, such as pancreatic ductal adenocarcinoma, glioblastoma, and colon carcinoma.^11–13^ According to TCGA database, ARNTL is differentially expressed in ESCC. However, the roles of ARNTL in ESCC development remain unclear.

Dual-specificity protein phosphatase-1 (DUSP1) is a member of the DUSP family, which is a group of phosphatases capable of dephosphorylating tyrosine or serine/tyrosine residues. It plays a crucial role in regulating the mitogen-activated protein kinase (MAPK) signaling pathway.^14^ MAPK pathway serves as a key mechanism for transducing extracellular signals to control essential cellular processes such as growth, proliferation, and apoptosis.^15^ The MAPK family contains three prominent members, namely extracellular signal-regulated kinase (ERK), c-Jun NH_2_-terminal kinase (JNK), and p38. Bioinformatics analysis has shown that DUSP1 might be a target of ARNTL. Thus, we hypothesized that ARNTL regulates DUSP1 to influence the activity of the MAPK pathway in ESCC.

In this study, we aimed to analyze ARNTL expression status and its downstream targets through bioinformatics analysis and sequencing. We intended to explore the interaction between ARNTL and DUSP1/ERK signaling. Furthermore, we sought to investigate the functions of ARNTL and DUSP1 in ESCC progression in vitro and in vivo. The findings of this study may provide a novel target for the treatment of ESCC.

## Materials and methods

### Bioinformatics analysis

ARNTL expression in esophageal carcinoma was analyzed using TCGA database and GTEx data through the GEPIA web server, containing 286 normal and 182 tumor samples.^16^ Moreover, the association between ARNTL and the histological types of esophageal carcinoma was analyzed by examining its levels in adenocarcinoma or squamous cell carcinoma according to TCGA database. The ARNTL enrichment analysis was performed through Gene Set Enrichment Analysis (GSEA) using the GSEA software,^17^ and results at *p* < .05 were considered statistically significant. The differentially expressed genes in ESCC were analyzed using the expression profiles in the GSE20347 and GSE45670 datasets, with the cutoff values of *p* ≤.001 and log fold change ≤ −1.

The binding sites of ARNTL on the DUSP1 promoter were predicted using JASPAR (https://jaspar.genereg.net/).^18^ Furthermore, DUSP1 targets were predicted using the Comparative Toxicogenomics Database (CTD; http://ctdbase.org/),^19^ and CTD-related pathways were analyzed through KEGG analysis using the DAVID tool (https://david.ncifcrf.gov/).^20^

### Cell culture and transfection

KYSE150 and TE-1 cells were obtained from Procell (Wuhan, China) and cultured in RPMI-1640 medium (Servicebio, Wuhan, China), containing 10% FBS (Servicebio) and 1% penicillin/streptomycin (P/S; Servicebio), at 37°C and 5% CO_2_. The 293T cell line was cultured in DMEM (Servicebio) with 10% FBS and 1% P/S.

The overexpression plasmid of human ARNTL (pLV3-CMV-ARNTL, also named ARNTL-ov) was obtained from Miaoling Biotechnology (Wuhan, China). The siRNAs targeting ARNTL (si-ARNTL-1: 5′-GGACCAGAGAAUGGACAUUUCCUdTdT-3′, si-ARNTL-2: 5′-GGAUGGCUGUUCAGCACAUGAAAdTdT-3′, and si-ARNTL-3: 5′-GCCAACAUUUCUAUCAGAUGACGdTdT-3′) and siRNA negative control (si-NC: 5′-UUCUCCGAACGUGUGCACGUdTdT-3′) were synthesized by Wuzhoukangjian Biological Technology Co. Ltd. (Tianjin, China). shRNAs targeting DUSP1 were inserted into the pLKO.1-PURO plasmid and synthesized by Qingke Biotechnology (Beijing, China). These vectors or siRNAs were transfected into cells with lipidosome 3000 (Thermo Fisher Scientific, MA, USA) or lipidosome 8000 (Beyotime, Shanghai, China). Cells transfected with the same vector without the insert were considered the control group.

### Quantitative reverse transcription polymerase chain reaction (qRT-pcr)

After isolation using RNA Extraction Kit (Servicebio), total RNA was used for cDNA synthesis using a cDNA Synthesis Kit (Servicebio), followed by qRT-PCR using SYBR® Select Master Mix (Thermo Fisher Scientific). The primers for ARNTL and DUSP1 are listed in Table 1. β-actin was used as a reference, and relative ARNTL and DUSP1 levels were calculated using the 2^−ΔΔCt^ method.

**Table 1.** Primers sequences used for qRT-pcr.

Gene name		Primers for PCR (5’-3’)
ARNTL (ENST00000389707.8-exon)	Forward	TGGGGCTGGATGAAGACAAC
	Reverse	CACCCTGATTTCCCCGTTCA
DUSP1 (ENST00000239223.4-exon)	Forward	AAGCAGAGGCGAAGCATCAT
	Reverse	CTGTTCGTGGAGTGGACAGG
β-actin (ENST00000493945.6-exon)	Forward	CTCGCCTTTGCCGATCC
	Reverse	TCTCCATGTCGTCCCAGTTG

### Cell viability assay using CCK-8 and calcein-AM/PI staining

In the CCK-8 assay, cells were dispersed in 96-well plates (1 × 10^4^/well), subjected to the indicated transfection, and cultured for 48 h. This was followed by incubation with 10 μL CCK-8 reagent (Beyotime) for another 2 h. The absorbance at 450 nm was measured using a microplate reader (Thermo Fisher Scientific). Cell viability was shown as a percentage (%) of the control or vector group according to the absorbance value. In the calcein-AM/PI double staining assay, cells were stained with the calcein-AM/PI working solution after 48 h of transfection, following the instructions of the Staining Kit (Beyotime). Through fluorescence microscopy (Keyence, Osaka, Japan), images in each sample were obtained, and the living cells were counted and analyzed.

### Cell colony formation and EdU proliferation assays

The influence of ARNTL or DUSP1 in ESCC cell growth was evaluated through standard colony formation and EdU assays. For cell colony formation analysis, ESCC cells at 48 h post-transfection were seeded in 12-well culture plates. Under standard growth conditions, the cells were allowed to grow for 10–14 days until colony formation. Following crystal violet (0.5%) staining, colony number (colonies with > 50 cells) were counted. For analysis of proliferation, ESCC cells after 48 h transfection were processed using EdU incorporation assay with the BeyoClick^TM^ EdU Kit and Alexa Fluor 488 as described by the manufacturer (Beyotime). The EdU-positive cells (green) were determined using a Keyence fluorescence microscope and plotted as a percentage (%) of total nuclei with blue fluorescence.

### Immunofluorescence assay

For cell immunofluorescence assay, KYSE150 and TE-1 cells were subjected to fixation and permeation after 48 h of the indicated transfection. Following 3% BSA blocking, cells were evaluated for PCNA expression by probing with an anti-PCNA antibody (GB11010, Servicebio), followed by incubation of Alexa Fluor 488-labeled IgG antibody (GB25303, Servicebio). For tumor immunofluorescence staining, the tumor tissues were fixed using 4% paraformaldehyde and paraffin-embedded and subsequently sectioned into 5 μm slices. These sections were deparaffinated, rehydrated, and treated with 1% Triton X-100 and 3% H_2_O_2_, followed by immersing in 3% BSA. Subsequently, the sections were incubated overnight with primary antibodies against PCNA (GB11010, Servicebio) or Ki67 (GB111499, Servicebio) and Cy3-conjugated secondary antibodies (GB21303, Servicebio) for 1 h. In both experiments, the nuclei were incubated with DAPI, and the sections were observed under a fluorescence microscope.

### Flow cytometry

Cell apoptosis was assessed through a flow cytometric technique using a CytoFLEX flow cytometer (Beckman Coulter, Brea, California, USA). KYSE150 and TE-1 cells were incubated with a solution containing 195 µL binding solution, 5 µL Annexin V-FITC, and 10 µL propidium iodide (PI) for 20 min in the dark, following the instructions of the Annexin V-FITC/PI Assay Kit (Beyotime). The apoptosis rate was scored using the FlowJo_V10 software.

### Western blotting

Protein was isolated using RIPA buffer (Beyotime) and quantified using a BCA kit (Beyotime). Subsequently, 20 μg of protein was separated via SDS-PAGE and transferred to PVDF membranes (Millipore, Bedford, MA, USA), and the membranes were immersed in 5% nonfat milk. They were then incubated overnight at 4°C with rabbit primary antibodies against DUSP1 (ab138265, Abcam, Cambridge, UK), BAX (GB114122, Servicebio), BCL2 (GB113375, Servicebio), ERK (GB11560, Servicebio), phosphorylated (p)-ERK (GB113492, Servicebio), ARNTL (14268–1-AP, Proteintech), pro-caspase 3 (ab32150, Abcam), cleaved-caspase 3 (ab32042, Abcam), p-p38 (AF1111, Beyotime), p38 (AF5890, Beyotime), JNK (17572–1-AP, Proteintech), p-JNK (80024–1-RR, Proteintech), and ACTB (GB11001, Servicebio) and at room temperature for 1 h with anti-rabbit secondary antibody (GB23303, Servicebio). The blots were visualized using an ECL kit (Servicebio) and analyzed using the Image J software.

### Xenograft experiments

Ten 5-week-old male BALB/c nude mice were obtained from Jiangsu Aniphe Biolaboratory Inc. (Nanjing, China). The TE-1 cells stably expressing ARNTL or control mock were constructed by infecting the cells with the relevant lentiviruses using standard protocols recommended by Qingke Biotechnology. They were then resuspended in phosphate buffer saline, followed by subcutaneous inoculation into mice. The mice were categorized into the ARNTL-ov lentivirus or vector group (*n* = 5). Animal health and behavior were monitored every three days. Tumor volume was monitored and calculated using the following formula: (length × width^2^)/2. After 5 weeks of cell implantation, all mice (*n* = 10) were anesthetized using 5% isoflurane within 5 min and then euthanized by cervical dislocation. The survival rate of the mice was 100%, and all mice died after euthanasia. Thereafter, the dissected tumors were weighed and used for the analyses of PCNA and Ki67 expression. Animal experiments were approved by the Animal Care and Use Committee of Henan Provincial People’s Hospital. All experimental procedures were performed in accordance with the international guidelines.

### RNA-sequencing (RNA-seq) analysis

RNA was isolated from TE-1 cells transfected with si-NC or si-ARNTL using RNA Extraction Kit (Servicebio). For RNA-seq, RNA was enriched via magnetic beads with Oligo (dT) and fragmented. The double-stranded cDNA was synthesized, and 3′-poly(A) tails and adapters were added to the cDNA ends. After the establishment of the cDNA library and purification, the paired-end sequencing reactions were conducted on the BGISEQ-500 sequencing platform (MGI Tech Co. Ltd., Shenzhen, China). Clean reads were obtained after filtering the low-quantity reads and adapters. The Cufflinks software was used for the determination of the fragments per kilobases per million fragments values. Differentially expressed genes were analyzed using DEGseq2 and were considered significant at *p* ≤ .001.

### Chromatin immunoprecipitation (ChIP)

The cell lysates of TE-1 cells were used for the ChIP assay Kit (Beyotime). The chromatin lysates were obtained via ultrasonication, and ChIP analysis was conducted using anti-ARNTL (ab230822, 1:200 dilution, Abcam) or IgG (ab205718, 1:1000, Abcam). After immunoprecipitation, the DNA level of DUSP1 was measured.

### DNA pull-down assay

The biotinylated DUSP1 promoter probe containing motif binding sites of ARNTL was synthesized by Qingke Biotechnology. They were then incubated with cell lysates for 8 h, followed by enrichment using streptavidin magnetic beads (Beyotime). After the washing, ARNTL enrichment levels on the bead were analyzed using a western blot assay.

### Luciferase reporter assay

The sequences of the DUSP1 promoter containing the wild-type (wt) motif binding sites (TCACGTG) of ARNTL were amplified using PCR with the following primers: forward 5′-GTAGTGTGGTTCTGGGCAAGTC-3′ and reverse 5′-CGCGTTTATATGCGGCCTCT-3′. These amplified sequences were then inserted downstream of the pGL3-basic vectors (Miaoling Biotechnology). The mutant (mut) luciferase reporter vectors were constructed by mutating the binding sites to GACATGT. The constructed wt or mut DUSP1 luciferase reporter vectors together with ARNTL-ov or empty vectors were transfected in 293T cells. After 48 h, luciferase activity was analyzed using a luciferase reporter assay kit (Promega Corporation, Madison, WI, USA) with pRL-TK (Miaoling Biotechnology) as a reference.

### Statistical analysis

The experiments were performed in triplicate, and results are presented as mean ± SD. Statistical analysis and graphs were conducted via GraphPad Prism 8 (GraphPad Inc., La Jolla, CA, USA). The comparison for two groups was performed using Student’s t-test, and comparison between multiple groups was conducted via one-way ANOVA, followed by Tukey’s test. Statistical significance was set at *p* < .05.

## Results

### Differentially expressed ARNTL is associated with ESCC progression

**Figure 1.**
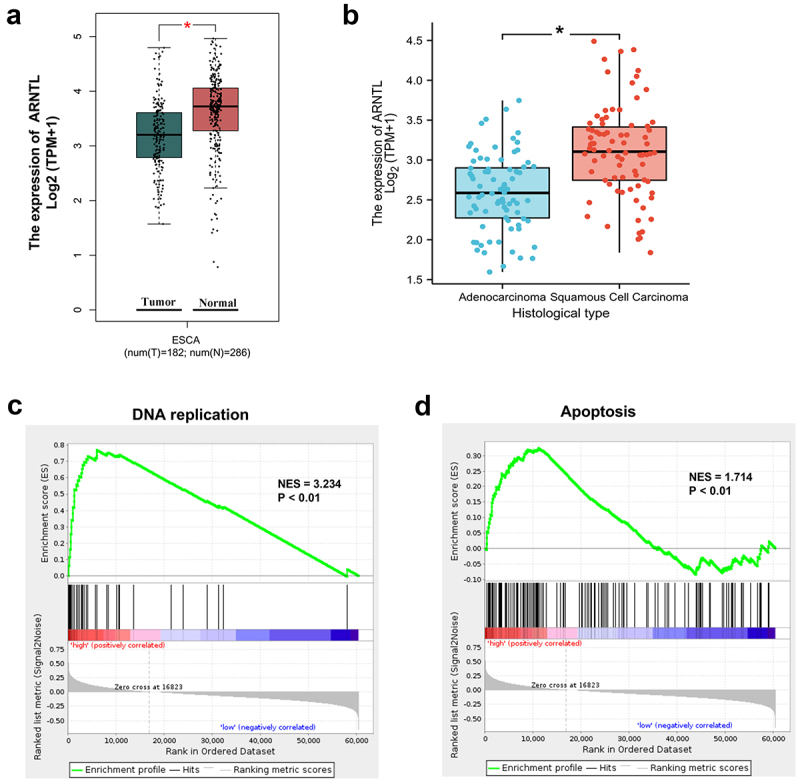
ARNTL is differentially expressed in ESCC.

To explore whether ARNTL was associated with ESCC progression, its expression was analyzed in esophageal carcinoma using TCGA database. ARNTL expression was observed to be markedly reduced in esophageal carcinoma compared with that in normal samples (Figure 1a). Moreover, ARNTL was significantly differentially expressed in ESCC and esophageal adenocarcinoma (Figure 1b). As ESCC is the primary histological type of esophageal carcinoma, we aimed to determine the functions of ARNTL in ESCC. The ARNTL-related pathways in ESCC were analyzed through GSEA, indicating that ARNTL was associated with DNA replication (NES = 3.234, *p* < .01) and apoptosis (NES = 1.714, *p* < .01; Figure 1c,d). These results suggested that ARNTL was associated with ESCC progression.

(a) ARNTL expression in esophageal carcinoma (ESCA) was analyzed according to TCGA database by the GEPIA webserver. (b) ARNTL expression in esophageal adenocarcinoma or squamous cell carcinoma was analyzed according to TCGA database. (c and d) ARNTL-related pathways were analyzed by GSEA. *p < .05.

### ARNTL impedes cell growth and promotes cell apoptosis in ESCC

**Figure 2.**
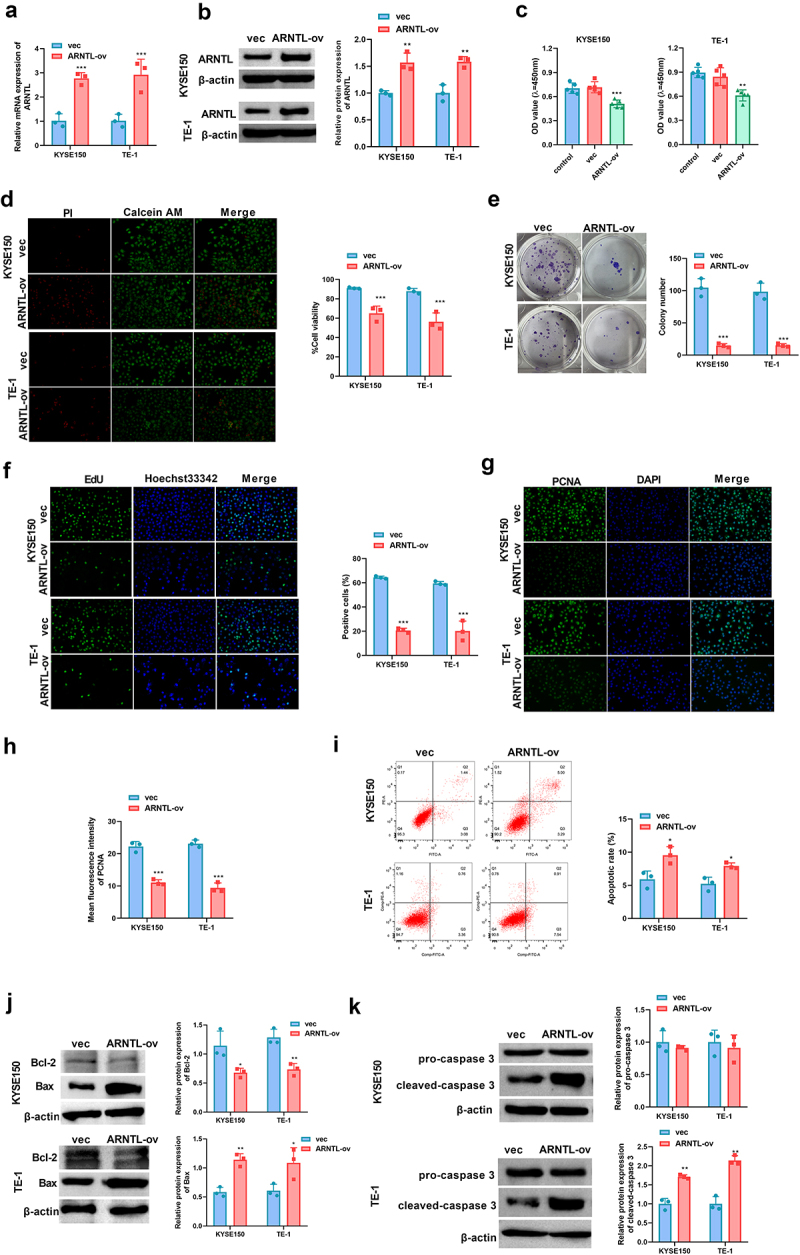
ARNTL upregulation hinders cell growth and promotes cell apoptosis in ESCC.

To assess the function of ARNTL in ESCC progression, its expression was analyzed and found to be elevated in ESCC cells. ARNTL mRNA and protein levels were elevated in KYSE150 and TE-1 cells after transfection with ARNTL-ov (Figure 2a,b). CCK-8 and calcein-AM/PI staining assays showed that ARNTL overexpression reduced cell viability (Figure 2c,d). Moreover, increased expression of ARNTL repressed cell growth and downregulated PCNA in ESCC cells (Figure 2e–h). ARNTL upregulation triggered the apoptosis of KYSE150 and TE-1 cells (Figure 2i), which was validated by the detection of apoptosis-related factors Bax, Bcl-2, and cleaved-caspase 3. Western blotting results revealed that an increase in ARNTL levels induced upregulation of Bax and cleaved-caspase 3 and downregulation of Bcl-2 (Figure 2j,k). These results indicated that elevated ARNTL expression facilitated ESCC cell apoptosis.

(a and b) ARNTL mRNA and protein levels were detected in KYSE150 and TE-1 cells transfected with vector or ARNTL-ov via qRT-PCR and Western blot. (c-k) KYSE150 and TE-1 cells were transfected with vector or ARNTL-ov, and then cell viability was detected by CCK-8 and Calcein AM/PI staining assays (c and d), cell growth was assessed by colony formation and EdU assays (e and f), PCNA expression was analyzed by immunofluorescence (g and h), apoptosis was measured using flow cytometry analysis (I), and the protein levels of Bcl-2, Bax, pro-caspase 3, and cleaved-caspase 3 were evaluated by western blot (j and k). Compared with vec, *p < .05; **p < .01; ***p < .001.

### ARNTL reduces ESCC cell growth in murine models

**Figure 3.**
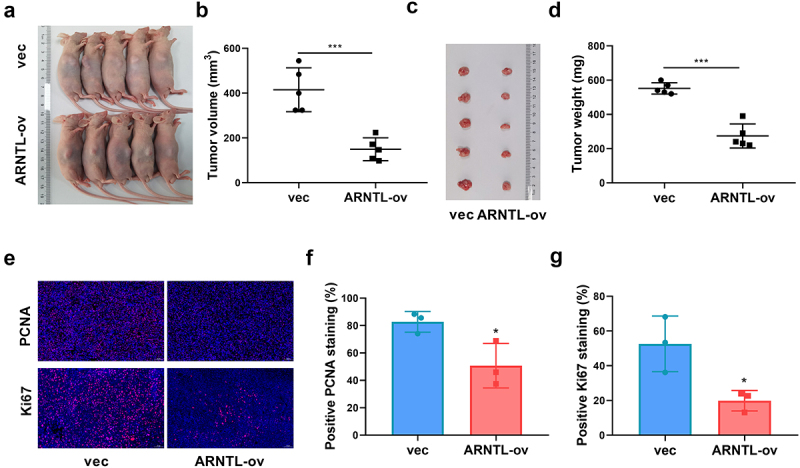
ARNTL upregulation reduces ESCC cell growth in murine model.

To further analyze the function of ARNTL in ESCC in vivo, the effects of ARNTL on ESCC tumorigenesis were assessed in xenograft models. TE-1 cells stably expressing ARNTL were used, and mice were categorized into vec or ARNTL-ov lentivirus group. After 5 weeks, the tumor volume and weight were markedly decreased in the ARNTL-ov group compared with those in the vec group (Figure 3a–d). Moreover, the immunofluorescence assay showed that PCNA and Ki67 were significantly downregulated in tumor tissues of the ARNTL-ov group compared with those in the vec group (Figure 3e–g). These results suggested that increased expression of ARNTL inhibited ESCC xenograft growth.

TE-1 cells stably transfected with vector or ARNTL-ov were injected into nude mice. (a) The images of mice with xenograft tumors in each group. (b) Tumor volume was detected. (c) The images of tumors. (d) Tumor weight was measured. (E-G) PCNA and Ki67 levels were detected by immunofluorescence assay. Compared with vec, *p < .05; ***p < .001.

### ARNTL knockdown decreases DUSP1 expression in ESCC cells

**Figure 4.**
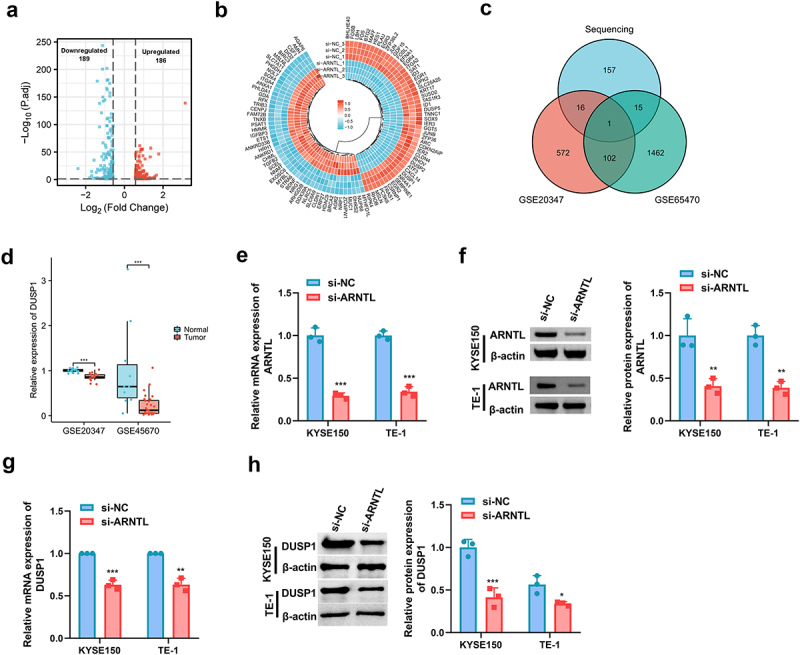
The potential targets of ARNTL.

To explore the potential downstream genes of ARNTL in ESCC, RNA sequencing analysis was performed on TE-1 cells with ARNTL silencing. In total, 375 genes exhibited significant differences in expression levels (*p* ≤.05, |log2FoldChange| > 0.58) following si-ARNTL transfection; here, 186 genes were upregulated and 189 were downregulated (Supplementary Table S1). The volcano plots and cluster heat map of differentially expressed genes are presented in Figure 4a,b). Moreover, we screened the downregulated genes using the sequencing data and differentially expressed genes in ESCC using the GSE20347 and GSE45670 datasets (*p* ≤ .001, log fold change ≤ −1; Supplementary Table S2). Through this matching process, *DUSP1* emerged as the only overlapping gene (Figure 4c). Reduced DUSP1 expression was observed in ESCC based on the GSE20347 and GSE45670 datasets (Figure 4d). High silencing efficiency of si-ARNTL was validated by RT-qPCR and western blot assays (Figure 4e,f). DUSP1 levels were significantly decreased because of ARNTL silencing (Figure 4g,h). These data showed that ARNTL could positively regulate DUSP1 in ESCC cells.

(a and b) Volcano plots and heat map of differentially expressed genes in TE-1 cells after ARNTL silencing. (c) The overlapping genes by matching sequencing data and datasets of GSE20347 and GSE45670. (d) DUSP1 levels in GSE20347 and GSE45670 datasets. (e and f) ARNTL mRNA and protein levels were detected in KYSE150 and TE-1 cells transfected with si-NC or si-ARNTL via qRT-PCR and western blot. (g and h) DUSP1 mRNA and protein levels were measured in KYSE150 and TE-1 cells transfected with si-NC or si-ARNTL via qRT-PCR and western blot. Compared with normal or si-NC, *p < .05; **p < .01; ***p < .001.

### ARNTL positively regulates the transcriptional activity of DUSP1 in ESCC

**Figure 5.**
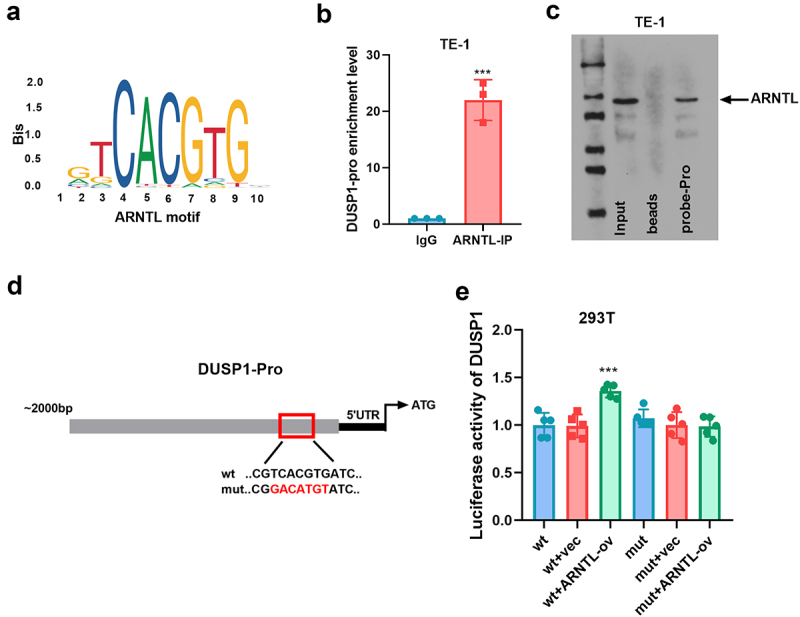
ARNTL regulates DUSP1 transcription.

To elucidate the mechanism through which ARNTL regulates DUSP1 in ESCC, we investigated the interaction between them. The motif of ARNTL is presented in Figure 5a. The association between ARNTL and DUSP1 was analyzed using ChIP and DNA pull-down assays, revealing that ARNTL could bind to DUSP1 in TE-1 cells (Figure 5b,c). The binding sites of ARNTL on the DUSP1 promoter are shown in Figure 5d, and the wt or mut luciferase reporter vectors were constructed. The luciferase reporter assay showed that the luciferase activity was markedly increased by ARNTL overexpression in 293T cells with transfection of wt-pGL3-DUSP1 vectors. This effect was abolished in the mut group, confirming that DUSP1 was targeted by ARNTL (Figure 5e). These results indicated that ARNTL could regulate DUSP1 transcription in ESCC cells.

(a) The motif of ARNTL. (b) The interaction between ARNTL and DUSP1 by ChIP assay. (c) The binding relationship between ARNTL and DUSP1 by DNA pull-down assay. (d and e) The luciferase activity was analyzed in 293T cells transfected with vector or ARNTL-ov together with wt-pGL3-DUSP1 or mut-pGL3-DUSP1. Compared with vec, ***p < .001.

### DUSP1 silencing reverses the effects of ARNTL on ESCC progression

**Figure 6.**
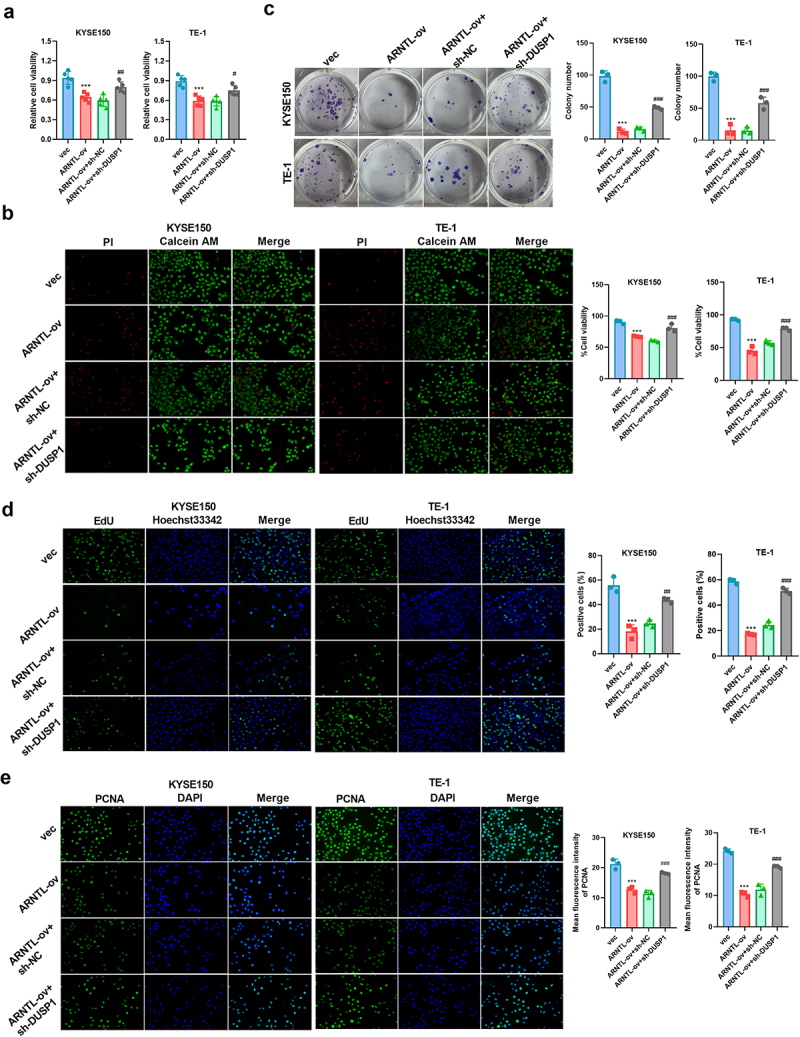
DUSP1 knockdown reverses the effect of ARNTL upregulation on cell growth in ESCC cells.

**Figure 7.**
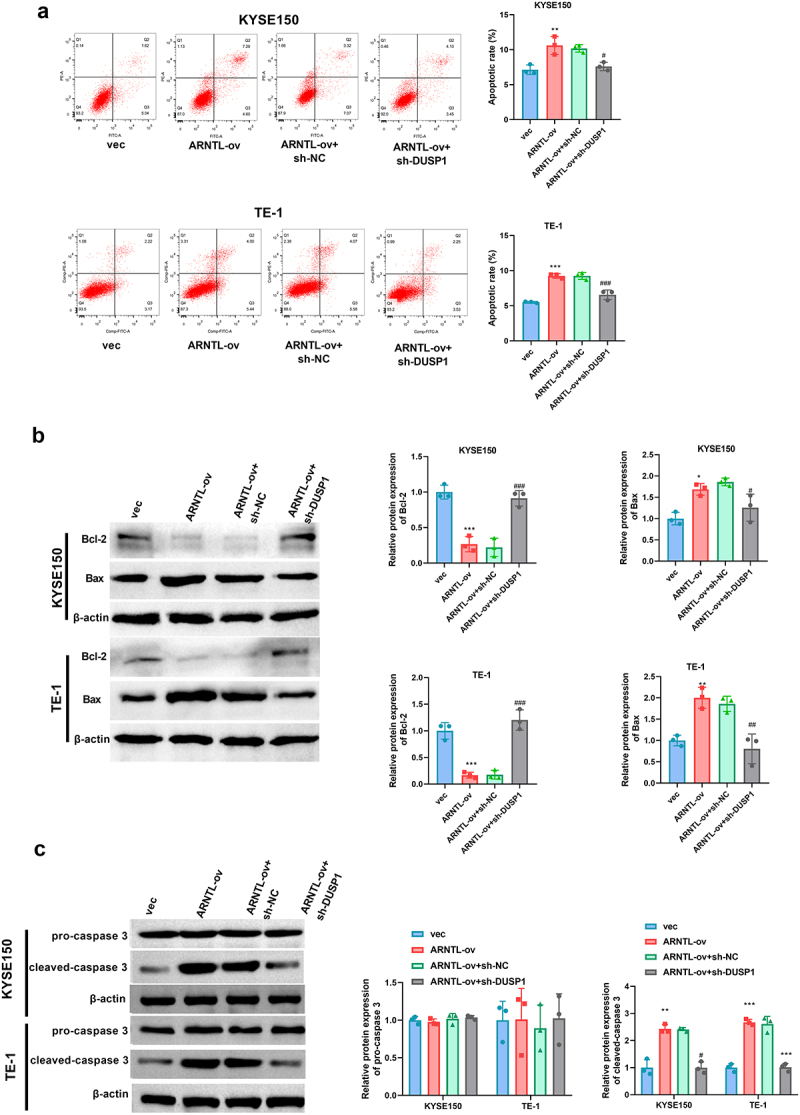
DUSP1 knockdown reverses the effect of ARNTL upregulation on cell apoptosis in ESCC cells.

To explore whether DUSP1 was associated with ARNTL-mediated ESCC progression, the effects of DUSP1 downregulation on ARNTL-mediated ESCC cell apoptosis were investigated. DUSP1 silencing by sh-DUSP1 transfection was found to reverse ARNTL-modulated reduction of viability, growth, and PCNA expression in KYSE150 and TE-1 cells (Figure 6a–e). Conversely, downregulation of DUSP1 reversed ARNTL-modulated promotion of apoptosis and alterations in Bcl-2, Bax, and cleaved-caspase 3 levels in the two ESCC cell lines (Figure 7a–c). These results showed that ARNTL regulated ESCC cell viability and apoptosis by modulating DUSP1.

(a-e) KYSE150 and TE-1 cells were transfected with vector, ARNTL-ov, ARNTL-ov + sh-NC, or ARNTL-ov + sh-DUSP1, followed by detection of cell viability by CCK-8 and Calcein AM/PI staining assays (a and b), cell colony formation by colony formation assay (c), cell proliferation by EdU assay (d), and PCNA expression by immunofluorescence (e). Compared with vec, ***p < .001; Compared with ARNTL-ov + sh-NC, ^#^*p* < .05; ^##^*p* < .01; ^###^*p* < .001.

(a-c) KYSE150 and TE-1 cells were transfected with vector, ARNTL-ov, ARNTL-ov + sh-NC, or ARNTL-ov + sh-DUSP1, followed by evaluation of cell apoptosis by flow cytometry assay (a), and Bax, Bcl-2, pro-caspase 3, and cleaved-caspase 3 levels by western blot (b and c). Compared with vec, **p < .01; ***p < .001; Compared with ARNTL-ov + sh-NC, ^#^*p* < .05; ^##^*p* < 0.01; ^###^*p* < .001.

### ARNTL inhibits activation of the ERK signaling by regulating DUSP1 in ESCC

**Figure 8.**
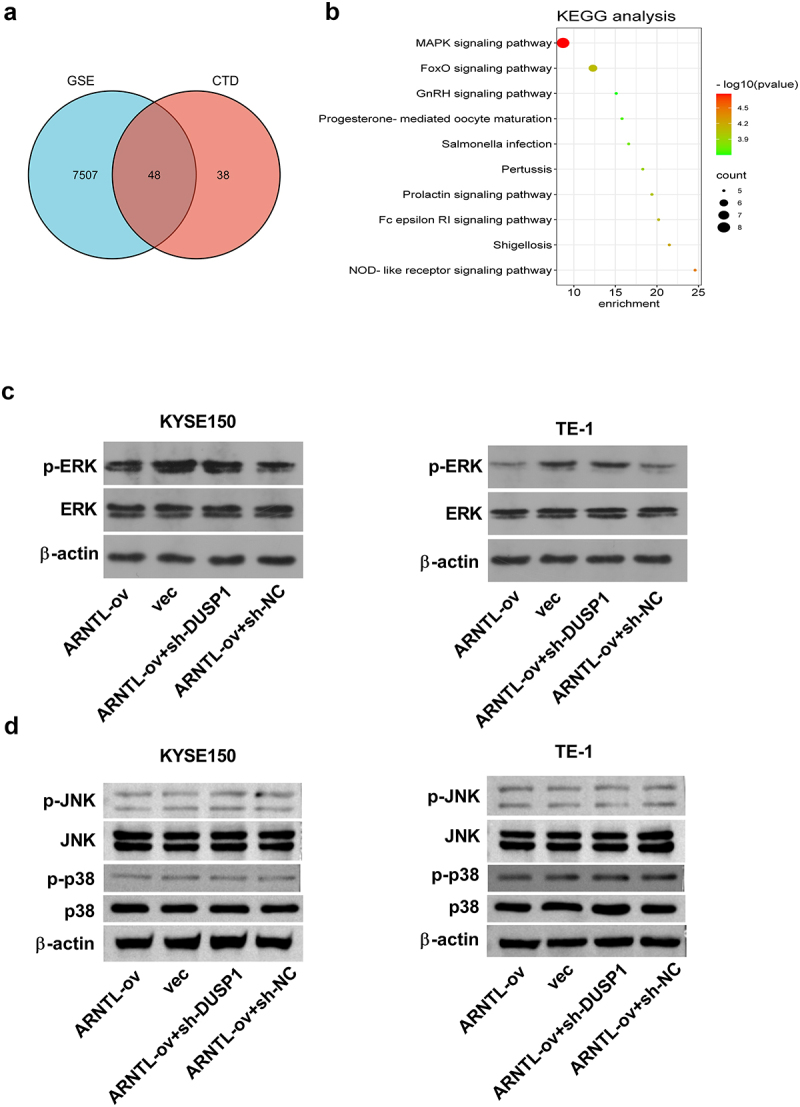
ARNTL upregulation blocks activation of the ERK signaling by upregulating DUSP1.

To further explore the downstream pathways governed by DUSP1, we analyzed the potential targets of DUSP1 using the CTD database (Supplementary Table S3). By matching these targets with differentially expressed genes in ESCC according to the GSE20347 and GSE45670 datasets (*p* ≤.001), we identified a total of 48 overlapped targets (Figure 8a). KEGG analysis was performed using the 48 targets using the DAVID web server. Results indicated that DUSP1-related targets might be associated with several pathways, including the MAPK signaling pathway (Figure 8b). We observed that the level of p-ERK was markedly decreased after ARNTL overexpression, which was largely reversed by DUSP1 silencing (Figure 8c). Meanwhile, western blot assay results revealed that the ARNTL-DUSP1 axis had no significant effect on the phosphorylation levels of JNK and p38 (Figure 8d). These findings indicated that ARNTL upregulation might inactivate the ERK signaling by regulating DUSP1.

(a) The DUSP1-related targets in ESCC by matching ARNTL targets (CTD) and differentially expressed genes in GSE datasets (GSE20347 and GSE45670). (b) DUSP1-related pathways were analyzed by KEGG analysis using the overlapping targets. (c and d) KYSE150 and TE-1 cells were transfected with vector, ARNTL-ov, ARNTL-ov + sh-NC or ARNTL-ov + sh-DUSP1. (c) p-ERK/ERK levels were detected by western blot. (d) The phosphorylation and total levels of JNK and p38 were determined by western blot.

## Discussion

ESCC is the most common type of esophageal carcinoma with high mortality worldwide.^21^ The transcription factors are linked to several diseases such as cancers, including ESCC.^22,23^ In this study, we aimed to explore the function of the transcription factor ARNTL in ESCC. We found that increased expression of ARNTL inhibits ESCC progression by hindering cancer cell growth and promoting cell apoptosis. Moreover, we verified that it may be associated with DUSP1-mediated inactivation of the ERK signaling.

Apoptosis is a key process in the anti-cancer treatment of ESCC.^24^ Multiple studies have validated the pro-apoptotic roles of ARNTL in human tumor cells, including glioblastoma, melanoma, tongue squamous cell carcinoma, and pancreatic cancer.^12,25–27^ Consistent with these reports, our study found decreased ARNTL expression in ESCC and confirmed that its upregulation could promote ESCC cell apoptosis and inhibit tumorigenesis in vivo. These findings indicate the therapeutic potential of ARNTL in ESCC.

To explore the downstream targets of ARNTL, we performed sequencing analysis in ESCC cells following ARNTL silencing. Through screening genes with reduced expression and matching them with downregulated genes in ESCC from the two GEO datasets (GSE20347 and GSE45670), DUSP1 was identified as a downstream target of ARNTL. This finding was further confirmed in our study, demonstrating that ARNTL positively regulates DUSP1 transcription in ESCC cells. Multiple studies have reported that DUSP1 might play an anti-tumor activity by inducing apoptosis in human tumors, including colorectal, breast, and prostate cancers.^28–30^ Similarly, our study identified the regulatory effects of DUSP1 on ESCC cell apoptosis. Moreover, DUSP1 silencing was found to attenuate the effects of ARNTL on ESCC progression, indicating that ARNTL regulated ESCC development by influencing DUSP1. DUSP1, a prominent member of the DUSP family, functions as a dual-specificity protein phosphatase capable of dephosphorylating both threonine/serine and tyrosine residues. The DUSP family is known to play a crucial role in regulating MAPK signaling pathways, with DUSP1 specifically targeting the following three members of MAPK families: JNK, p38, and ERK. Studies have indicated that DUSP1 exhibited specificity in dephosphorylating distinct MAPK pathways in various tumor types, such as targeting the JNK pathway in prostate cancer^31^ and the p38/MAPK pathway in hepatocellular carcinoma.^32^ However, the specific role of DUSP1 on MAPK subfamilies in ESCC remains to be determined. Here, our data showed that ARNTL attenuated ERK activity by reducing ERK phosphorylation through DUSP1 upregulation in ESCC cells. ERK activation is responsible for cell growth and metastasis in ESCC.^33,34^ Moreover, as the primary member of the MAPK family, ERK activation contributes to ESCC development.^33–35^ Hence, we hypothesized that ARNTL upregulates DUSP1 to induce ERK inactivation, thereby constraining ESCC progression in vitro.

In conclusion, upregulation of ARNTL impedes ESCC cell growth and facilitates cell apoptosis, partly by upregulating DUSP1 to induce ERK inactivation. Our study findings indicate the potential of ARNTL in the treatment of ESCC. Further animal studies are required to better understand the role of ARNTL in ESCC.

## Ethical approval

All animal experiments were performed with the approval of the Animal Ethics Committee of Henan Provincial People’s Hospital and the procedures for Care and Use of Laboratory Animals in cancer research.

## Supplementary Material

Supplementary Table 1.xlsx

Supplementary Table 3.xlsx

Supplementary Table 2.xlsx
